# Lipoprotein (a) and coronary heart disease – is there an efficient secondary prevention?

**DOI:** 10.1007/s11789-017-0088-x

**Published:** 2017-02-23

**Authors:** Klaus-Peter Mellwig, Dieter Horstkotte, Frank van Buuren

**Affiliations:** Clinic for Cardiology, Herz- und Diabeteszentrum NRW, Ruhr-Universität Bochum, Georgstr. 11, 32545 Bad Oeynhausen, Germany

**Keywords:** Lipoprotein (a), Lp (a), Coronary heart disease (CHD), Acute coronary syndrome, Intervention, Lipid apheresis, Prevention

## Abstract

Lipoprotein (a) (Lp (a)) is one risk factor for the development of cardiovascular diseases. Several studies have shown that Lp (a) hyperlipoproteinaemia has a particular influence on the development of coronary heart disease (CHD). A retrospective single-centre observation study was performed to evaluate the effectiveness of lipid apheresis on the basis of consecutively performed percutaneous coronary interventions (PCI) in patients with high Lp (a) values and angiographically documented CHD.

In 23 pts (male 18, age 60.04 ± 0.58 years) with angiographically documented CHD (first manifestation 48.00 ± 9.41 years), elevated LDL cholesterol (144.39 ± 92.01 mg/dl) and Lp (a) (133.04 ± 39.68 mg/dl), 49 PCI and 3 coronary artery bypass grafting (CABG) procedures had been performed prior to the initiation of lipid apheresis. Following the initiation of weekly lipid apheresis, LDL cholesterol was 99.43 ± 36.53 mg/dl and Lp (a) 91.13 ± 33.02 mg/dl. In a time interval of 59.87 ± 49.49 months (median 51.00, range 1–153 months) 15 pts did not require an additional PCI. In 8 pts (7 pts 3‑vessel disease, 1 pt 2‑vessel disease) 14 PCI – no CABG – were performed after 69.38 ± 71.67 months (median: 32.50, range 17–232 months). The incidence of PCI could thus be reduced by 71.43%.

## Introduction

Lipoprotein (a) (Lp (a)) is one of the most atherogenous lipoproteins and its impact on atherogenesis has become of increasing scientific interest during recent years [1]. Many studies have shown that elevated Lp (a) concentrations have an important influence on the development of coronary heart disease (CHD) [2–4]. The structure of Lp (a) consisting of the Lp (a)-specific glycoprotein apolipoprotein-a (APO (a)) implies the pro-atherogenous and pro-thrombogenous characteristics [3] with increased risk of myocardial infarction and heart failure [5].

Our retrospective study in 31,734 patients over a 5-year period documented that an increasing Lp (a) concentration is associated with a higher incidence of CHD and severity of vascular disease (Table 1) as well as more myocardial infarctions, interventions and surgical myocardial revascularizations (Table 2).

**Table 1:** Incidence of coronary vessel disease associated with increasing Lp (a) levels

Group	1 VD (%; *n*)	2 VD (%; *n*)	3 VD (%; *n*)
Lp (a) ≤ 2 mg/dl	10.3%; 22	8.5%; 18	9.9%; 21
Lp (a) 23–29 mg/dl	15.7%; 50	12.2%; 39	25.1%; 80
Lp (a) 30–60 mg/dl	15.6%; 35	9.8%; 22	27.1%; 61
Lp (a) 61–90 mg/dl	23.1%; 53	17.5%; 40	40.2%; 92
Lp (a) 91–110 mg/dl	19.2%; 30	19.9%; 31	38.5%; 60
Lp (a) > 110 mg/dl	14.0%; 37	18.2%; 48	50.4%; 133

*VD* vessel disease

**Table 2:** Incidence of myocardial infarction, PCI and CABG under increasing Lp (a) levels

Group	Myocardial infarction (%; *n*)	PCI (%; *n*)	CABG (%; *n*)
Lp (a) ≤ 2 mg/dl	11.8%; 25	22.2%; 47	9.0%; 19
Lp (a) 23–29 mg/dl	16.6%; 53	33.6%; 107	20.8%; 66
Lp (a) 30–60 mg/dl	18.2%; 38	56.6%; 125	26.4%; 58
Lp (a) 61–90 mg/dl	26.9%; 61	78.5%; 179	33.5%; 76
Lp (a) 91–110 mg/dl	35.7%; 55	72.3%; 112	30.1%; 47
Lp (a) > 110 mg/dl	34.4%; 90	54.8%; 144	40.5%; 107

Current options of drug therapy are very limited. A significant reduction of Lp (a) concentrations is neither achieved by statins or ezetimibe nor by PCSK9 inhibitors [6, 7]. In contrast, high doses of nicotinic acid (niacin) (2–4 g) significantly reduce Lp (a) [8]. However, since 2013 this substance is not available any more in Europe due to its side effects.

Lipid apheresis seems to be the only therapeutic option providing a favourable effect on cardiac events and the progression of coronary heart disease.

## Patients and methods

In a retrospective, single-centre observation study, the effectiveness of lipid apheresis in patients with high Lp (a) levels and angiographically documented coronary heart disease was evaluated on the basis of percutaneous coronary intervention (PCI). All patients undergoing lipid apheresis during the observation period with Lp (a) > 60 mg/dl prior to the first procedure were included. All patients fulfilled the criteria of the German federal authority to be eligible for lipid apheresis, and reimbursement had been approved by committees of regional associations of statutory health insurance physicians. Furthermore, all patients gave their informed consent to the scientific analysis of their data.

Twenty-three patients (male 18, age 60.04 ± 10.58 years) fulfilled the above criteria and were included in the study (Table 3). Apheresis was performed applying the heparin-induced extracorporeal LDL precipitation (HELP) (Plasmat-Futura®, B. Braun, Melsungen, Germany) and the polyacrylate adsorption of whole blood or plasmapheresis by means of double filtration (DALI® and MONET®, Fresenius Medical Care, Bad Homburg, Germany). During the weekly procedures, lipid status (Lp (a), total cholesterol, HDL cholesterol, LDL cholesterol, triglycerides) was obtained before and after the procedure. Reduction of LDL cholesterol and Lp (a) was 60–65% per procedure.

**Table 3:** Patient characteristics

Patients (*n*)	23
Men (*n*)	18
Women (*n*)	5
Age (y) (at the time of the study)	60.04 ± 10.58
Men (y)	61.11 ± 8.45
Women(y)	54.75 ± 14.72
Age (y) (first manifestation of CHD)	48.00 ± 9.20
Men (y)	50.00 ± 7.81
Women (y)	40.80 ± 10.19
BMI (kg^.^m^−2^)	26.33 ± 4.08
***Coronary status***	*Pat (n/%)*
0-vessel disease	1/4.34
1-vessel disease	5/21.73
2-vessel disease	3/13.04
3-vessel disease	14/60.87
*Vascular morphology (segm. occlusion)*	*Pat (n/%)*
1 vessel	5/21.73
2 vessels	2/8.68
3 vessels	1/4.34
**PCI pre**	49
*Number of PCI*	*Pat (n/%)*
5 x PCI	1/4.34
4 x PCI	2/8.68
3 x PCI	5/21.73
2 x PCI	7/30.43
1 x PCI	7/30.43
0 x PCI	1/4.34
CABG prior to lipid apheresis	3/13.04
**PCI post**	14
*Number of PCI*	*Pat (n/%)*
1 x PCI	5/21.73
2 x PCI	1/4.34
3 x PCI	1/4.34
4 x PCI	1/4.34
CABG following lipid apheresis	0
LV dysfunction	Pat (*n*)
LV-EF: <0.50	5/21.7
*Peripheral arterial occlusive disease*	*Pat (n/%)*
Angiographically proven	5/21.7
Intervention	3/13.0
*Cerebral atherosclerosis*	*Pat (n/%)*
Symptomatic Insult	1/4.3
Carotid stenosis (haemodynamically not relevant)	5/21.7
*Arterial hypertension*	*Pat (n/%)*
Drug therapy	16/69.6
*Chronic renal insuffiency (eGFR)*	*Pat (n/%)*
≥90 (ml/min)	13/56.52
60–89 (ml/min)	8/34.78
30–59 (ml/min)	2/8.69

### Lipid status

Prior to the initiation of lipid apheresis, LDL cholesterol was 144.39 ± 92.01 mg/ld and Lp (a) 133.04 ± 39.68 mg/dl. 18 patients received the highest tolerated dosage of statins, 8 patients of them ezetimibe in addition. 5 patients had a statin intolerance.

### Cardiovascular status

Fourteen of 23 patients had a coronary 3‑vessel disease (3-VD), 3 patients a 2-VD, 5 patients a 1-VD. One patient with a history of a TIA turned out to suffer from coronary sclerosis (0-VD). In 5 patients, CHD was associated with segmental occlusions and another 5 patients showed an additional involvement of the vascular periphery and the cerebrovascular region (Table 3). Patient age at first disease manifestation was low with 48.00 ± 9.41 years, in 10 patients first manifestation was accompanied by an acute coronary syndrome (ACS).

Prior to lipid apheresis, 49 PCI and 3 CABG procedures had been performed in patients with a haemodynamically relevant coronary stenosis. Lipid apheresis was initiated after a mean duration of 4.30 ± 4.95 years following first manifestation of CHD. Thereafter, all patients underwent non-invasive diagnosis on a routine basis every year and invasive diagnostic procedure every 2 years followed by PCI in case of a haemodynamically relevant stenosis (Table 3).

## Results

**Fig. 1:**
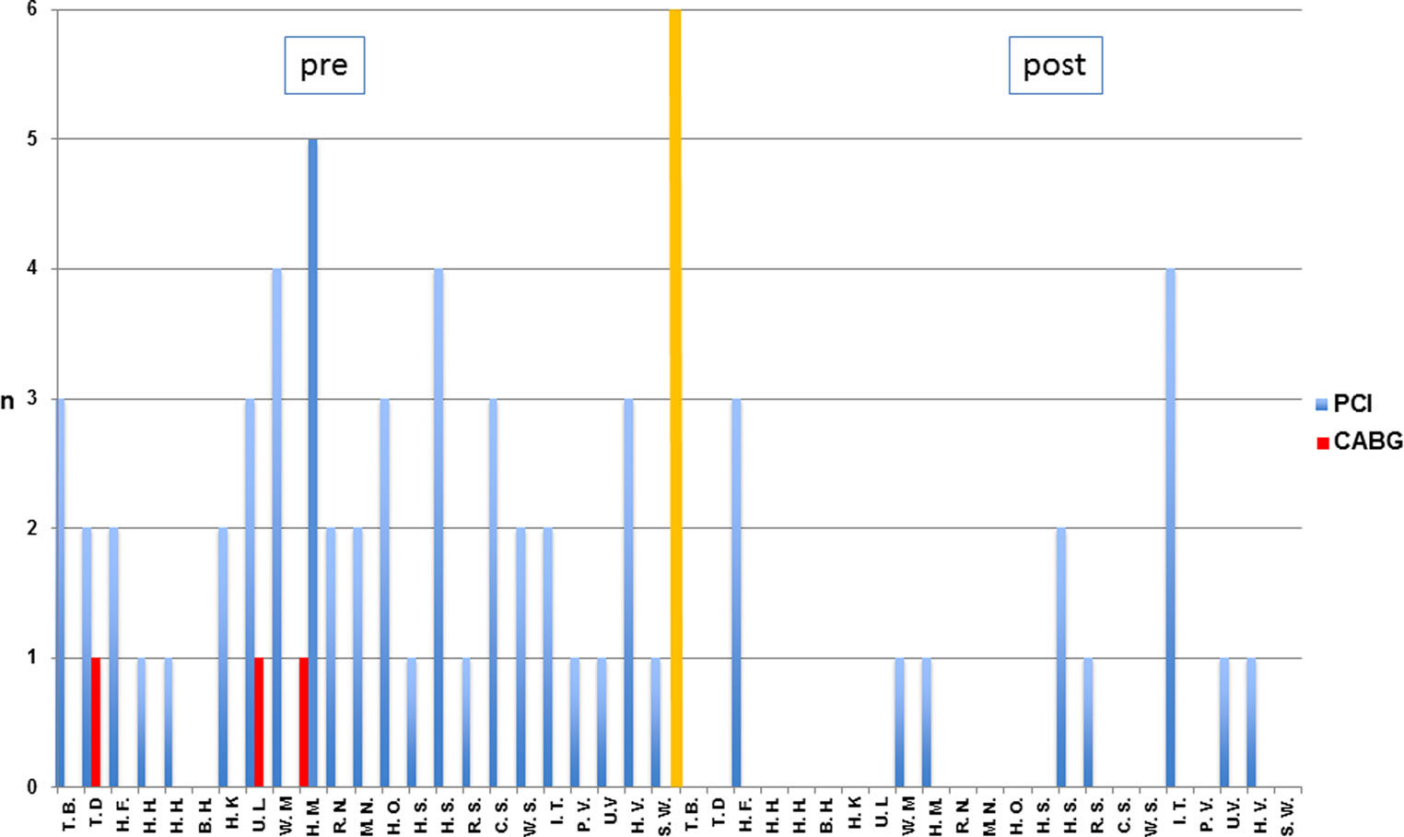
Interventions (PCI/myocardial revascularization) before and after the initiation of lipid apheresis

At the time of the study, LDL cholesterol was 99.43 ± 36.53 mg/dl and Lp (a) 91.13 ± 33.02 mg/dl. In a time interval of 59.87 ± 49.49 months (median 51.00, range 1–153 months) 15 patients did not require a PCI. For the first time after initiation of lipid apheresis, 8 patients (7 pat 3‑VD, 1 pat 2‑VD) underwent PCI after 69.38 ± 71.67 months (median: 32.50, range 17–232 months) the total number of PCI being 14. Two of 3 patients with recurrent stenosis and multiple interventions had an in-stent restenosis. CABG was not necessary (Fig. 1).

## Discussion

The present study was intended to document the influence of lipid apheresis on the incidence of PCI. The patient cohort with a high Lp (a) concentration was characterized by a diffuse, advanced CHD with a high proportion of segmental coronary occlusions. The additional high proportion of patients with cerebrovascular and peripheral vascular deterioration shows the vascular manifestations to be expected.

Regular lipid apheresis significantly reduced LDL cholesterol and Lp (a). In contrast to the multicentre prospective study evaluating cardiac events (MACE) after the initiation of lipid apheresis [9], our collective underwent PCI after documentation of a haemodynamically relevant stenosis as a sign of major disease progression. The significant decline in PCI procedures (71.43%) indicates the impact on the atherosclerotic process mainly induced by Lp (a) hyperlipoproteinaemia. This is of particular importance as it is a diffuse vascular disease of different vascular origin with a strong tendency to progression. At present, lipid apheresis has to be regarded as the most effective therapeutic procedure in secondary prevention.

## Conclusion

Our single centre observation study in patients with markedly increased LDL cholesterol and Lp (a) values accompanied by an early manifestation of coronary heart disease and consecutive PCI or surgical myocardial revascularization showed that lipid apheresis reduced the incidence of PCI or surgical myocardial revascularization by 71.43%. This therapeutic option has to be part of secondary prevention.
